# Parent Perspectives on Physical Therapy for Their Child with Acute Lymphoblastic Leukemia: *The Light at the End of the Tunnel*

**DOI:** 10.3390/curroncol33010060

**Published:** 2026-01-20

**Authors:** Paula A. Ospina, Palana Shah, Livleen Dhaliwal, Sara Fisher, Beverly A. Wilson, Lesley Pritchard, David D. Eisenstat, Margaret L. McNeely

**Affiliations:** 1 Department of Physical Therapy, Faculty of Rehabilitation Medicine, College of Health Sciences, University of Alberta, Edmonton, AB T6G 2G4, Canada; pospina@ualberta.ca (P.A.O.); palana@ualberta.ca (P.S.); ldhaliwa@ualberta.ca (L.D.); lwiart@ualberta.ca (L.P.); 2 Pediatric Oncology Unit, Stollery Children’s Hospital, Edmonton, AB T6G 2B7, Canada; sara.fisher@albertahealthservices.ca (S.F.);; 3 Department of Pediatrics, Faculty of Medicine and Dentistry, University of Alberta, Edmonton, AB T6G 2R3, Canada; 4 Children’s Cancer Centre, Royal Children’s Hospital, Parkville, VIC 3052, Australia; 5 Department of Pediatrics, University of Melbourne, Parkville, VIC 3052, Australia; 6 Department of Oncology, Faculty of Medicine and Dentistry, University of Alberta, Edmonton, AB T6G 2R3, Canada; 7 Cancer Care Alberta, Alberta Health Services, Edmonton, AB T5J 3E4, Canada

**Keywords:** physical therapy, children, cancer, barriers, facilitators

## Abstract

Many children with cancer referred to physical therapy (PT) do not attend the service. We conducted a pilot study, comprising a cross-sectional survey and interviews with parents of children with acute lymphoblastic leukemia. The survey explored parents’ (1) views on PT service delivery for their child, (2) perspectives on barriers and facilitators, (3) preferred timing to introduce PT, and (4) views on virtual services. Questions were designed based on the Theoretical Domains Framework, and responses were mapped onto the Capability, Opportunity, Motivation–Behavior Change Model. Twenty parents participated in the survey. Although all parents would consider their child accessing PT if deficits were present, access depended on a convenient location (70%) and availability of virtual delivery (45%). While half of the parents preferred PT treatment to be introduced during the maintenance phase of chemotherapy, findings also support earlier introduction during the consolidation phase when services are framed as part of standard care. While most parents perceived that it would be manageable to support home-based PT, barriers included a lack of child’s motivation without therapist support. Seven parents participated in semi-structured interviews. They identified time constraints, distance, and costs as common barriers. Most parents responded positively to hybrid PT models and connections with community locations to mitigate these challenges.

## Simple Summary

Physical therapy (PT) services for children with cancer are limited, and children often do not attend their appointments. We conducted a cross-sectional survey and semi-structured interviews to better understand parents’ perspectives towards PT services for children with acute lymphoblastic leukemia. Twenty parents completed the survey. All parents were willing for their child to access PT if physical challenges were present; however, factors such as a convenient location and availability of virtual delivery were necessary to facilitate access to the service. Seven parents participated in semi-structured interviews. Time constraints, distance, and costs were common barriers identified. Parents highlighted the importance of a PT approach tailored to the child’s physical condition, abilities, and family context. Most parents had a positive response to virtual delivery and connections with community PT locations to mitigate barriers to access rehabilitation services. Understanding parents’ perspectives is key to improving access of children with cancer to PT services.

## 1. Introduction

Advances in cancer treatments and earlier diagnosis of acute lymphoblastic leukemia (ALL) have increased the 5-year survival rate for Canadian children to 94% [1]. However, improved survival has also led to a growing number of children living with long-term side effects from cancer treatments [2]. Children with ALL treated with chemotherapy agents (e.g., vincristine, intrathecal methotrexate) and steroids (e.g., dexamethasone) have a high risk of developing neuromuscular and musculoskeletal side effects such as muscle weakness [3,4], impaired balance [5,6], range of motion deficits [3,4,7,8], impaired gross-and fine-motor performance [4,9,10,11], impaired gait [12], decreased energy expenditure [13], and chemotherapy-induced peripheral neuropathy [14,15].

While some deficits resulting from ALL therapies may improve following treatment, many persist or worsen. Functional mobility upon completion of cancer treatment remains below age norms, with 5% to 54% of childhood cancer survivors continuing to experience deficits [4,10,16,17]. Literature shows that children with ALL present with decreased passive and active range of motion in ankles and hips [3,4,17,18] and impaired walking capacity [3,8] years after chemotherapy completion. Sequelae from ALL treatment regimens may lead to long-term deficits that impact physical function [3] and contribute to frailty in adulthood, which is associated with a new onset of chronic health conditions at a younger age [19]. Therefore, children with ALL require close monitoring and timely intervention to address cancer therapy side effects, as they can persist or develop months or years after completion of cancer treatments [20].

Comprehensive cancer care in children must include the management of short- and long-term deficits resulting from ALL treatments. Physical therapy (PT) can help children with ALL maximize functional mobility and develop motor skills, minimizing the sequelae to increase children’s participation in daily life activities [21] and improving quality of life across the cancer continuum of care [22,23,24]. However, few childhood cancer survivors access pediatric oncology rehabilitation services [25,26,27,28]. Barriers for families of children with cancer include geographical distances, time constraints, and the absence of childcare options for their other children [29]. In 2017, we conducted a Canada-wide survey exploring the referral practices and healthcare providers’ perspectives on the barriers to access pediatric oncology rehabilitation services. Healthcare providers indicated that the main reason children and adolescents with cancer did not receive rehabilitation services was related to family choice [28]. This finding was supported by a retrospective chart review and a prospective questionnaire conducted by Gohar et al., exploring the frequency and rationale behind physicians’ referral of children with ALL to PT services [30]. Parent choice and financial resources were primary reasons children with ALL did not receive rehabilitation. To date, little is known about the needs and perspectives of parents of children with ALL towards PT intervention, so healthcare providers know what areas must be addressed in care.

### Objectives

To explore parents’ perspectives regarding (1) their capacity, skills, means, motivation, and interest to access and support PT service delivery for their child; (2) the factors that hinder and/or enable their child’s participation in PT programs; and (3) the best time to introduce a PT intervention for their child.

## 2. Materials and Methods

### 2.1. Study Design

The study involved a mixed-methods research design comprising quantitative and qualitative data. We conducted a pilot study, comprising a cross-sectional survey, followed by semi-structured interviews to explore the perspectives of families of children with ALL related to PT services.

#### 2.1.1. Conceptual Framework

The development of the cross-sectional survey was guided by the Theoretical Domains Framework (TDF) [31] and the Capability, Opportunity, Motivation—Behavior Change (COM-B) Model [32]. Questions focused on understanding the perspectives and experiences of parents of children with ALL to inform the design of tailored PT programs. The TDF is an integrated theoretical framework designed to aid application of theoretical approaches to behavior change interventions [31]. The TDF comprises 14 domains and 84 constructs to allow for the synthesis of multiple behavior change theories into a single framework, facilitating the assessment of behavioral problems, barriers, and facilitators, and informing the design of appropriately tailored interventions [31,32].

The COM-B Model was designed to assist in the identification of appropriate targets for behavior change [33]. The COM-B contains three main factors: (1) capability: refers to the individual’s psychological and physical capacity to engage in the behaviors or activities concerned; (2) opportunity: is defined as all factors external to the individual that influence the behavior; and (3) motivation: refers to the brain processes that energize and direct behavior [32]. Each domain of the TDF can be mapped to a component of the COM-B Model, allowing for a systematic approach to interpretation of findings and identification of actionable strategies for improving PT access and engagement.

The survey included a total of 17 questions (15 single/multiple-choice and 3 open-ended) (Table 1 and Document S1). Survey responses were mapped to the COM-B Model; areas identified for further exploration were addressed in the semi-structured interviews.

#### 2.1.2. Qualitative Methodology

We applied an interpretive descriptive qualitative methodological approach to understand parents’ perspectives and experiences with PT services for their child [34]. This approach is well suited to clinical health research, especially in areas where empirical evidence is limited and where further practical, reflexive clinical knowledge is instrumental for building a meaningful theoretical foundation for inquiry [34,35]. Interpretive Description was selected as it is designed to provide insight into the impact of human health and disease experiences on health practices [34], aligning with the study aims and consistent with the scope of the inquiry.

### 2.2. Sampling and Participants

A convenience sample of parents of children diagnosed with ALL at the Stollery Children’s Hospital in Edmonton, Alberta, who were undergoing or who had completed ALL therapy and consented to receive information about the study, were contacted. All children with ALL at the hospital are monitored by PT services as per standard of care protocol. We estimated a sample size of 20 survey participants, based on the average number of children receiving ALL therapy at the hospital per year (~30).

Individuals were eligible for the study if they were (1) a parent/primary caregiver of a child diagnosed with ALL aged between 4 and 17 years (at time of diagnosis), undergoing or having completed ALL therapy, and (2) able to speak and comprehend English. We aimed to include families with children at different phases of cancer treatment to collate perspectives across the cancer trajectory.

Parents were identified at the pediatric oncology clinic upon referral from the oncology team and were provided with information about the study. Parents interested in the study were contacted to discuss the research study and answer any questions. Upon completion of screening for eligibility, written consent was provided. Following enrollment in the study, participants received a link to a Research Electronic Data Capture (REDCap) service, a secure electronic data capture tool hosted and supported by the Women and Children’s Health Research Institute at the University of Alberta [36], to fill out a demographic information questionnaire and the survey.

After consenting to the study, parents were provided with the option to participate in semi-structured interviews. Given the burden of cancer in families, as well as the challenges in talking about a sensitive topic, interview participation was dependent on the availability and interest to engage. The interview sample was not intended to be representative but rather to capture a range of perspectives [34]. A target sample of 6 to 8 parents/primary caregivers was selected to allow for variability in perspectives while supporting in-depth qualitative analysis. Consistent with qualitative methodology, the sampling approach allowed for the possibility of expanding the data collection process [34].

### 2.3. Data Collection

The survey was open from July 2022 to February 2023. Participants were provided the option to complete the survey online at home or on paper at one of their child’s appointments at the hospital. Parents willing to participate in the semi-structured interviews were contacted approximately four months following the survey completion to book the semi-structured interview. Sessions were conducted in person at the next hospital visit or through a secure Zoom™ (Edmonton, AB, Canada) videoconference call at their preferred time.

Results from the cross-sectional survey served to identify key areas for further exploration during the semi-structured interviews. Two study investigators reviewed the data and identified the TDF domains and components of the COM-B Model that suggested divergent viewpoints or those that demanded further input from parents (P.A.O. and M.L.M.). The two investigators designed questions addressing the identified areas that required further exploration through semi-structured interviews (P.A.O. and M.L.M.). The interviews were approximately 60 min long, facilitated by a member of the research team (P.A.O.), and audio recorded in Zoom™, using the audio transcription feature. Data collection continued until confidence was reached that the findings were sufficiently developed and no new themes emerged, indicating thematic saturation [34].

### 2.4. Data Analysis

Quantitative data collected from the survey were analyzed descriptively and presented as means ± standard deviation (SD), medians, frequency (f), interquartile range (IQR), and/or percentage. Two study investigators coded responses from open-ended questions, and results were reported as frequencies (P.A.O. and M.L.M.).

For the semi-structured interviews, one member of the research team (P.A.O.), verified all the transcriptions verbatim. Data were analyzed using an inductive thematic analysis process, following the six phases of analysis described in Braun & Clarke 2022 [37], to identify patterns and themes. Four team members (P.A.O., P.S., L.D. and M.L.M.) examined each transcript independently to code the data, identify patterns, create themes, and select key quotations supporting the statements shared by participants. Each member independently reviewed all interview transcripts multiple times to become familiar with the data and to generate initial codes. Codes were developed inductively and linked to data extracts. Coder triangulation was achieved through iterative refinement of codes, first individually and then through group discussion to resolve discrepancies and to fully represent the meaning of participants’ voices. Team members reviewed the codes and inductively generated themes and sub-themes by sorting the codes into potential themes, with the use of visual representations to facilitate the process. We reviewed and refined the themes for each participant’s data set and for the entire data set [38]. Given that this type of analysis is not linear but rather an iterative and recursive process involving a constant comparison across analytic phases [38], allowing the opportunity to pause and reflect on our notes. Finally, qualitative data was mapped back to the TDF domains and components of the COM-B model.

The credibility of the research findings was supported through transparency in the analytic reasoning process throughout the study [34]. The investigator facilitating the interviews kept a reflexive journal to record their research engagement and inter-subjective experiences throughout the study and shared notes with other team members to address potential biases, challenges, and analytic processes [39]. Rigor was enhanced through thick descriptions and the extensive use of direct quotes from participants, rather than providing opinions on the information gathered [39]. Since investigators only had access to the participants’ ID, this helped remove the bias of associating the responses to an individual.

### 2.5. Ethical Considerations

Ethics approval was granted by the Health Research Ethics Board of Alberta (HREBA)—Cancer Committee (CC). All parents/primary caregivers provided written informed consent and were assigned a study number. Steps were taken to protect the confidentiality of participants and to ensure unbiased responses. First, to further protect the identity of participants, all identifiable data were removed from information related to the interviews (e.g., child’s name). Second, to minimize potential bias in the survey and interview questions, a priori feedback was obtained from clinicians, parents, and investigators on the clarity and transparency of the questions and interview prompts, as well as to avoid leading questions.

## 3. Results

**Figure 1:**
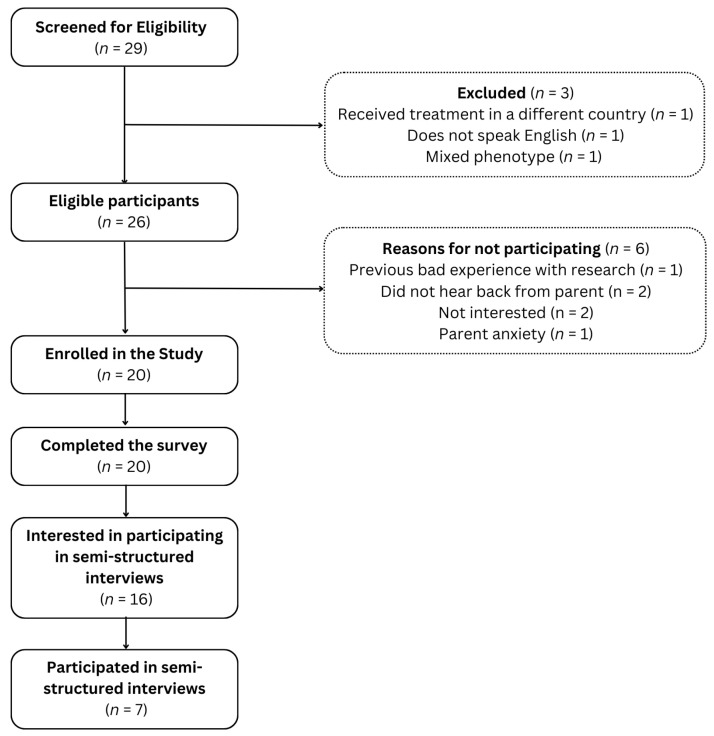
Flowchart of recruitment.

A total of 26 parents of children undergoing or having completed ALL treatment were eligible to participate in the study, and 20 provided written consent to participate in the survey. Of the 20 parents, 16 consented to be approached to participate in the semi-structured interviews, and seven consented to participate (Figure 1). Results from the survey and interviews are presented in two separate sections.

### 3.1. Survey Results

A large proportion of parents were identified as being female (*n* = 15, 75%). Over 50% of parents had completed a university/college degree. Geographical location of residential living varied among participants, with 55% of parents living in the Edmonton Metropolitan Area and 45% in other locations within and nearby the province. The mean age of parents’ children at the time of the survey was 7.3 years (range 4–14 years). The majority of participants (*n* = 16, 80%) were parents of children undergoing the maintenance phase of ALL treatment—reflecting the usual clinic demographics (Table 2).

Survey results were summarized by the three components of the COM-B model: Capability, Opportunity, and Motivation.

#### 3.1.1. Capability

Most parents reported being familiarized with the benefits of PT services for their child (*median= 72.50 (IQR: 50–87); distribution based on direction: ‘not at all’ to ‘very much’*). All parents considered it important to access PT services if their child was experiencing difficulties with their physical ability (*n =* 20/100%).

While 95% of parents said they would consider accessing PT for their child to help reduce treatment-related side effects and maintain their physical function during cancer treatment, opinions were divided regarding the preferred timing for their child to start a PT program. Fifty-five percent of parents indicated they would prefer the PT program to start during the maintenance phase of ALL treatment (last phase), 35% from the induction phase (first phase), 5% from diagnosis, and 5% from the consolidation/intensification phases (middle phases).

**Figure 2:**
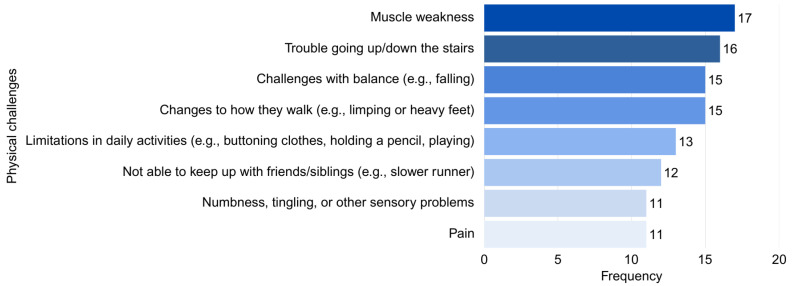
Physical challenges that may prompt parents to seek physical therapy for their child.

Reported difficulty accessing PT services varied. Forty-five percent of parents reported being neutral (not easy or hard), 30% did not know, 20% reported PT was very easy to access, and 5% indicated PT was very difficult to access. The most common physical challenge that may prompt parents to seek PT for their child was muscle weakness (*f =* 17, 85%), followed by trouble going up/down the stairs (*f =* 16, 80%) (see Figure 2).

#### 3.1.2. Opportunity

The main resources identified as needed to be in place for parents to access PT services for their child included a ‘convenient/accessible location’ (*f =* 14, 70%) and ‘public or private healthcare coverage for costs of PT services’ (*f =* 14, 70%), followed by ‘time’ (*f =* 10, 50%) and ‘coverage to pay for indirect/other costs (e.g., parking, childcare, time off work)’ (*f =* 5, 25.0%). Correspondingly, the main factor that would influence parents’ decision to access PT services if the child requires it included ‘distance to PT service’ (*f =* 14, 70%), followed by ‘availability of options for PT delivery: home-based, virtual, in-person’ (*f =* 9, 45%), ‘personal income’ (*f =* 8, 40%), ‘expertise of physical therapist’ (*f =* 7, 35.0%), ‘research evidence supporting PT interventions’ (*f =* 2, 10.0%), and ‘child’s current health status’ (*f =* 1, 5%).

The majority of parents (*n* = 17, 85%) believed that their family or other immediate members would be supportive of their child accessing PT services. Two parents were unsure, and one parent believed others would not be supportive of their child accessing PT services.

#### 3.1.3. Motivation

Parents’ confidence in deciding whether their children needed PT treatment varied across participants. Half of the parents (50%) felt ‘very much’ confident, 30% felt ‘somewhat’ confident, 15% felt ‘undecided’, and 5% felt ‘not really’ confident.

When parents were asked how PT could help if their child was experiencing pain, difficulty walking, or muscle weakness, parents indicated that PT can help by prescribing exercises/treatment (*f =* 13), strengthening muscles (*f =* 9), stretching the body (*f =* 4), offering PT direction/advice/support (*f =* 5), offering opportunities for play (*f =* 2), providing pain management (*f =* 2), providing gait education (*f =* 1), providing movement resources (*f =* 1), prescribing orthotics (*f =* 1), and managing chemotherapy side effects (*f =* 1).

Most parents reported feeling ‘calm/confident’ if their child was having issues with activities such as walking or getting dressed and needed to be referred to PT services (*median= 83.50 (IQR: 73–99.25); distribution: ‘upset/concerned’ to ’calm/confident’*). Likewise, 75% considered that the benefits of PT for their child would justify the costs and time, while a few were unsure (25%).

Most parents indicated that the best way to support their child’s PT treatment was by ‘helping them with exercises at home’ (90%), followed by ‘arranging PT appointments’ (80%), ‘providing encouragement’ (80%), and ‘being there for them’ (5%).

The majority of parents (85%) considered the oncologist responsible for referring their child to PT services, 45% considered the nurse responsible, 35% the family doctor, 20% the teacher at school, 10% did not know, and 5% indicated hospital physical therapists should be responsible.

Most parents reported they would find it ‘easy’ *for their child to do exercises at home* (e.g., a home exercise program assisted by a parent) (*median= 21 (IQR: 0–50); distribution: ’very easy’ to ’very difficult’*). When looking at the reasons for ease of home exercise programs, many parents identified the availability of space and equipment (*f =* 8), availability of time (*f =* 2), child/parent motivation (*f =* 2), parental support (*f =* 1), availability of internet connection (*f =* 1), physically active child (*f =* 1), and flexibility with scheduling (*f =* 1) as factors that would enable the child to do a home-based exercise program. Barriers such as lack of motivation to exercise without the support of a therapist (*f =* 4), distractions at home (*f =* 2), child not being physically active (*f =* 1), lack of time (*f =* 1), and inability to troubleshoot potential issues at home (*f =* 1) were identified as factors that could hinder the child’s participation in a home-based program.

Similarly, most parents reported they would find it ‘easy’ *to support their child in doing exercises at home* (e.g., a home exercise program assisted by a parent) (*median= 27.5 (IQR: 0–50); distribution: ’very easy’ to ’very difficult’*). Parents reported several factors that would facilitate supporting their child to exercise at home, including the availability of parental support (*f =* 9), availability of time (*f* = 6), availability of space and equipment (*f* = 2), flexibility with scheduling sessions (*f* = 1), dedicated time to exercise (*f* = 1), and physically active family (*f* = 1). Barriers reported were the lack of parent availability (*f* = 4), lack of child’s motivation to exercise without the therapist’s support (*f* = 3), distractions at home (*f* = 1), lack of time (*f* = 1), and issues troubleshooting potential issues at home (*f* = 1).

### 3.2. Mapping of Survey Results into the COM-B Model

**Figure 3:**
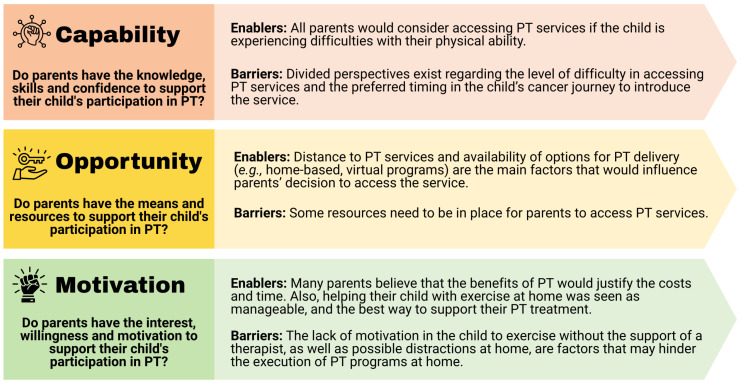
Mapping of survey results into the Capability, Opportunity, Motivation—Behavior Change (COM-B) Model. Abbreviations: PT, physical therapy.

Findings from the survey results were mapped into the COM-B model (Figure 3). Survey results with divided perspectives on certain areas, as well as topics that would benefit from further inquiry to explain survey findings or gather potential solutions, were covered in-depth through semi-structured interviews. Semi-structured interview questions were designed based on the COM-B components and survey topics identified that warranted further exploration, including parents’ perspectives on the (1) benefits and challenges they had experienced with PT services (*capability*), (2) best timing during their child’s cancer treatment to initiate PT (*capability*), (3) resources that need to be in place to access PT services for their child (*opportunity*), and (4) alternative modes of delivery and ideal PT locations (e.g., virtual PT) *(motivation)*. We also included a question on parents’ perspectives on a proposed hybrid PT program utilizing an online platform. Findings related to parents’ perspectives on the hybrid program will be published elsewhere (please refer to Document S2 to access the semi-structured interview questions).

### 3.3. Semi-Structured Interview Results

Of the seven parents who agreed to participate in the semi-structured interviews, four were parents of children undergoing the maintenance phase (last phase) of ALL treatment, two were parents of children undergoing the consolidation phase (middle phase) of ALL treatment, and one was a parent of a child who had completed ALL treatment.

Three themes, along with their corresponding sub-themes and supporting quotations to illustrate participants’ perspectives, were elicited from the semi-structured interviews:

**THEME 1: The Child and Family are an Interconnected Unit**

Parents emphasized the importance of introducing PT early as an integral part of cancer care, with relationship building to help families understand the role of PT in supporting the child’s function and recovery. They described the need for healthcare providers to adopt a family-centered approach to rehabilitation that considers the needs of the child, parent, and siblings. The child’s physical, emotional, and functional status was seen as closely linked to the parents’ ability to provide support and balance competing family responsibilities. Likewise, parents’ fears and priorities influenced both their own motivation and their child’s subsequent engagement in PT. This interdependent relationship underscores the need to consider both the child and family unit when planning and delivering rehabilitation services.

**Sub-theme 1.1: Shared Emotional Impact**

Physical limitations from medical procedures, side effects from cancer treatments, physical inactivity, and the burden of hospital appointments negatively affected children’s participation in daily life activities such as school, play, and sports. Parents worried that their child was falling behind compared to peers and shared the increased stress over uncertainty about the child’s cancer and treatment journey.

“*At the beginning [of the cancer journey] it was pretty scary. Obviously going through what we were going through…the cancer treatment alone. But then, [it] was difficult. But then, here you have a child that you know couldn’t walk. Like couldn’t do things that she should be able to do. And I think we were just really afraid that she would be so far behind everyone else her age*.”

(P19—Parent of a 7-year-old off ALL treatment)

The amount of new medical information given to parents, particularly at the beginning of the cancer journey, was perceived as overwhelming, limiting their ability to retain the information provided.

“*You definitely get a ton of information at the hospital and a ton of names thrown at you that you may not remember at the time. And then, there’s a lot of information overload. When you’re at the hospital, you know, doctors are saying: “Okay, you know, watch for this, watch for fever, watch for…” You know, like parents are thinking: “Fever? Oh, my God*”.”

(P1—Parent of a 7-year-old off ALL treatment)

The hospital environment and need for isolation were perceived as additional stressors to the child, impacting function and making the cancer journey more difficult.

“*[Child’s name]...a lot of the time when he was seen, [he was] in isolation. So, basically, nobody would come in… He was just inside his little room, that was it. So, basically, he walked to the bathroom, and then he walked back. And it was getting to the point that [child’s name] couldn’t even walk to the bathroom.*”

(P11—Parent of a 7-year-old undergoing the maintenance phase of ALL treatment)

Parents expressed that considering attending PT sessions sometimes felt overwhelming and trivial during cancer treatment. Parents highlighted the need for PTs to recognize what the family is going through at the time, even when communicating the importance of PT treatment.

“*There’s a time and place, and sometimes the physical therapists don’t realize when we’re not at that stage, right? I know that physiotherapy pushes like movement. But sometimes, if you’re in survival mode, that’s an extra stress you don’t need. So, I felt a little bit of pressure there. And... [child’s name] felt a little bit of pressure. I tried to shield him from it*.”

(P16—Parent of a 9-year-old undergoing the consolidation phase of ALL treatment)

Parents reported that their child often felt discouraged when they had performed poorly on the PT assessment tests or when exercises did not go well, prompting them to look for small achievements to celebrate.

“*I was impressed with what he did [during the physical therapy assessment], but I know he tells me afterwards how he feels about those [physical therapy] tests, and he knows he didn’t do as good as he should do, and that’s part of it. So, his bedroom is downstairs… he had to do stairs every day. That was like a big thing, right? And that should be seen as an accomplishment*.”

(P16—Parent of a 9-year-old undergoing the consolidation phase of ALL treatment)

**Sub-theme 1.2: Family Participation and Support**

Educational resources from the physical therapist helped parents to better understand their child’s condition and support their rehabilitation. This eased uncertainties and reduced the burden of care.

“*I think we just feel so helpless, right? Like we are just watching our little babies just struggle. So, it’s kind of nice to feel like we are actually doing something to help them.*”

(P2—Parent of a 5-year-old undergoing the maintenance phase of ALL treatment)

Opportunities for families to participate together through home-based or virtual programming that included siblings were seen as providing motivation and connection.

“*Her brother could have participated with her [in the virtual session] and that maybe would have given her a little bit more motivation to beat him or work a little harder to keep up with him. But also from a parent’s perspective, I could have gone and made supper while they were both entertained and doing this physio[therapy] program on the TV [television] in the living room. So, that could help both ways*.”

(P1—Parent of a 7-year-old undergoing the maintenance phase of ALL treatment)

“*If there are other kids at home we included [child’s name] older sister in some of the [activities]. Like, when we’re playing balloon badminton, we were all doing that. We would be dancing together. We would be coming up with like an obstacle course in our hallway, and it was like a play, like it was a family activity.*”

(P19—Parent of a 7-year-old off ALL treatment)

**Sub-theme 1.3: Balancing Competing Family Demands**

Parents described the challenge of meeting the needs of the child undergoing treatment while maintaining stability for other family members. Many noted that time, energy, and emotional capacity were stretched thin, making it difficult to consistently support PT sessions or home exercises. The demands of work, caring for siblings, and coordinating hospital appointments often required families to compromise on competing demands.

“*They (hospital staff) may work with my schedule, I appreciate [it]…that’s good for us. Yeah, if they can do that with all the parent[s], because I told you, because parent[s] beside[s] [of] take care of sick kids, have so many thing[s] out to worry, and if many trip[s] [to the hospital] in the week, make[s] them more stressful.*”

(P18—Parent of a 9-year-old undergoing the consolidation phase of ALL treatment)

Parents identified financial barriers such as service charges, hospital parking fees, and gas costs. The need for support, such as fully covered sessions as well as free and easily accessible parking at the hospital, was identified as a key factor that could improve access to care. Indirect costs, such as lost income from taking time off from work or expenses for alternate caregiving, further compounded the financial burden and contributed to increased stress for families.

“*So, the stress of always finding someone to care for the other children. The expenses, you know, when you’re not working. So, obviously, just like the driving, and the gas. And you know, then you want to take her out and get her lunch after and stuff like that.*”

(P2—Parent of a 5-year-old undergoing the maintenance phase of ALL treatment)

**THEME 2: PT as the ‘Light at the End of the Tunnel’**

A supportive family-physical therapist relationship was seen as conducive to creating an environment that reduced parental fear and motivated them to have their child engage in physical activity. The acknowledgement of small accomplishments by the physical therapist provided reassurance to families about the child’s progress and provided hope that the child would return to their expected age-appropriate level of function.

“*So, I think it [physical therapy] just allowed us, after we spent many months in the hospital, to kind of have a **light at the end of the tunnel**, and that was always the goal of the team. You will go back to being able to do what you can—[you] can’t see it—but you know, as they help you through to that goal.*”

(P9—Parent of a 14-year-old undergoing the maintenance phase of ALL treatment)

**Sub-theme 2.1: Physical and Mental Health Benefits**

Parents perceived that PT was beneficial in (1) identifying and addressing cancer treatment-related deficits promptly; (2) improving crucial components of physical fitness and well-being such as muscle strength, balance, and flexibility; and (3) relearning skills that are essential for improving physical function.

“*My husband and I both feel that keeping her active has been a huge part in her success. And I mean, mentally, it has helped her, and physically, it has helped her. And I mean, she never had to use a wheelchair or a stroller or anything. [Child’s name] cancer was a big part in her legs, and before we got a diagnosis she wasn’t walking already. She was already starting to slow down, so getting her moving again was huge, and I think it helped her mentally and physically*.”

(P1—Parent of a 7-year-old undergoing the maintenance phase of ALL treatment)

**Subtheme 2.2: Confidence, Self-efficacy, and Motivation**

Perceived additional benefits from PT treatments on the child included (1) increased confidence in participating in physical activities, (2) self-efficacy in overcoming treatment-related challenges, and (3) increased motivation to maintain an active lifestyle. Together, these mental and emotional benefits instilled a sense of “normalcy” for both the child and parent.

“*Her feet are improving, she’s having more fun, and she’s going outside. And it’s not disrupting play with friends, which is huge. So, she’s starting to feel more confident in herself and her friend making ability. And that’s huge for me.*”

(P2—Parent of a 5-year-old undergoing the maintenance phase of ALL treatment)

Parents perceived that PT helped children take part in sporting activities and engage with peers socially, resulting in continued participation in usual activities.

“*So, I think that [physical therapy] was hugely positive for [child’s name], and just having to be able to do the same thing right, and play the sport she loved, and be with the friends that she loves. So, I think, from a mental care side of it, even though it’s a physical thing. It was, you know, hugely important on the mental side of it, for all of us right, so that [child’s name] could continue you know.*”

(P9—Parent of a 14-year-old undergoing the maintenance phase of ALL treatment)

**THEME 3: Multiple Routes, One Destination**

Parents described the need for PT to be flexible and responsive to the unique circumstances of each child and family. There was no identified single pathway through rehabilitation; instead, parent comments suggest the importance of tailoring the approach to the child’s physical condition, age, treatment phase and abilities, and family context. Early education was seen to help families understand what to expect, identify problems early, and feel more capable in assisting with rehabilitation through the cancer journey. These accounts illustrate that effective PT in pediatric oncology likely requires individualized, context-specific approaches that meet families where they are—acknowledging that multiple routes can lead to the same goal of supporting the child’s function and recovery over time.

“*We just need, the parents, all the resources, and just need to have knowledge about it all. And then they [parents] can figure out what works […] So, knowing that we can do outpatient [physical therapy], knowing that [physical therapist’s name] [is] there, having like an online kind of tool that we can do also. And I think, also, being given the facts that I could do physio[therapy] in [my city], if I wanted.*”

(P2—Parent of a 5-year-old undergoing the maintenance phase of ALL treatment)

“*It [cancer journey] was very overwhelming, and you are bombarded with social workers and everything right? So, it is overwhelming. But I also feel like it [physical therapy] needs to all be talked about at the beginning, processed, and brought back up again. I think that the role of the experts is to come to the people whose lives have just been turned upside down and say: “This is what you require”. The oncologist doesn’t say: “Would you like to take the chemo[therapy]?” No, they say: “You are going to take the chemo[therapy]”. The physio[therapist] says: “You are going to need physio[therapy]”. The nutritionist says: “You are gonna need to eat”. I think that’s all very important, because you’re not in a frame of mind to make those decisions yourself. And if [physical therapy] is valued by the care team as something that’s going to help children get better and quicker; then to me, it’s a huge benefit. And like I said at the time, you’re not thinking.*”

(P9—Parent of a 14-year-old undergoing the maintenance phase of ALL treatment)

**Sub-theme 3.1: Education and Knowledge Upfront**

Parents appreciated discussing possible changes in the child’s physical function, increasing their awareness of what signs to expect or look out for throughout their child’s cancer treatment.

“*I think the biggest benefit is just to have the dialogue so that we know what to look out for and what’s normal and acceptable as a weakness. And what needs to be worked on.*”

(P16—Parent of a 9-year-old undergoing the consolidation phase of ALL treatment)

Parents also expressed the importance of assessing the family’s capacity alongside the occurrence and progress of side effects in the child. If side effects are present, then simple strategies could be suggested.

“*This tip is like really helpful, for you know, strengthening the back of the ankle, or whatever, and just like little movements. Because, like me, [I] just feel so lost. Like, I don’t know how to strengthen her ankle right? We feel completely helpless. So even if there were just like tips on like the parent side, that would be a relief. We can help them a little bit, even if it’s minor. It gives us some like power, knowing like we’re not helpless like with our child.*”

(P2—Parent of a 5-year-old undergoing the maintenance phase of ALL treatment)

**Sub-theme 3.2: Integrating PT into Daily Routines**

Parents identified the need for exercises that could be easily incorporated into daily living activities/routines, allowing for better adherence.

“*Like to incorporate that [physical therapy] in your daily routine with the family. So, it doesn’t necessarily need to take like an extra hour of time. [Let’s] say you’re going to the park with for your kids, so you can think of like, or we can give you exercises that you can incorporate to your routine.*”

(P16—Parent of a 9-year-old undergoing the consolidation phase of ALL treatment)

“*So like last time we went [to physical therapy], her feet are doing good, but she’s still struggling a bit. So, [physical therapist’s name] said to work on her core. So, even with like a 3-wheeled scooter, I was like: “That’s awesome! We don’t have that”. So, it’s those things with [physical therapist’s name], where she tells me things I can take home and implement, and they make a huge difference. So, [physical therapist’s name] [is] great at that. And that’s where physio[therapy] has really helped.*”

(P2—Parent of a 5-year-old undergoing the maintenance phase of ALL treatment)

**Subtheme 3.3: Tailoring Exercises to the Child**

Parents appreciated when physical therapists were attentive to subtle functional changes and designed tailored exercises that were both achievable and most helpful to the child.

“*I can say that, like [physical therapist’s name] is so great. So, we will do stuff over there [hospital], and then she will notice what part, or what muscle, or which foot needs to be worked on. And then, she will relay it to me. And then, she will give me ideas of what could help that.*”

(P2—Parent of a 5-year-old undergoing the maintenance phase of ALL treatment)

**Subtheme 3.4: Convenient Location and Flexible Scheduling**

Offering different location options for PT services (e.g., connection with locations in the community or school, or home visits) was seen to allow parents to choose based on priorities such as proximity to home or availability after school hours.

“*I didn’t want him to get sick or anything like that. So, he missed a lot of kindergarten. I would have like appreciated somebody coming into my house, like physio[therapy] coming to my house. Or even if there was like a [physical therapy] program we could do locally here, that was kind of overseen by pediatric oncology, but could be administered locally, we would absolutely make that happen for her if that’s what she needed*.”

(P11—Parent of a 7-year-old undergoing the maintenance phase of ALL treatment)

Flexibility with PT appointments that allowed last-minute rescheduling or weekend availability to accommodate for the family’s needs was appreciated. Families liked the convenience of having PT appointments coordinated with the child’s pre-existing hospital visits, minimizing travel and disruption to daily life.

*“[Coordinating physical therapy with hospital appointments is] even more beneficially, because you are already here. You can do it right after, or even before his appointment, whatever works better. That would be really, really beneficial for a lot of families.*”

(P11—Parent of a 7-year-old undergoing the maintenance phase of ALL treatment)

However, while convenient, in some cases, multiple hospital appointments in a day were also seen as difficult for the child.

“*If that [in-hospital physical therapy] is what [child’s name] needed, we would absolutely do that. For us, personally, being so far away it would work out better. But at the same time, we also have to balance it. Like, she is seeing a mental health counselor at the Stollery [Children’s Hospital] as well. So, when we go for oncology appointments, we try. I mean, we have always been able to schedule the same day. So, then, I mean, that might get full if she is seeing the mental health counselor, plus her doctors, plus going to the OR [operating room] for, and going for physiotherapy. So, it has to be a balance between all the different services.*”

(P1—Parent of a 7-year-old undergoing the maintenance phase of ALL treatment)

**Sub-theme 3.5: Fun, Play, Social**

Parents appreciated kid-friendly PT interventions that were tailored to the child’s needs and deficits, while being fun and adapted to the environment.

“*Obviously I don’t mind like, I want her to have me as an aid and stuff, but it [virtual exercise] was all very like regimented. And I think if it [virtual exercise] was more kind of play, and she didn’t need me, or like I’m still there, but if it was more loosely like, kind of like, fun.*”

(P2—Parent of a 5-year-old undergoing the maintenance phase of ALL treatment)

“*Even within the hospital room, to get the kids out of bed. One day she [physical therapist] just brought some bean bag animals, they [physical therapist and child] hid them around the room, and they hide and seek with these animals. And I mean it was pretty low impact, but it got [child’s name] out of bed for like half an hour, and then she gets tired. And then, like an hour later, she’d want to go back to hiding these animals and playing hide and seek again.*”

(P1—Parent of a 7-year-old undergoing the maintenance phase of ALL treatment)

## 4. Discussion

This pilot mixed-methods study explored the perspectives of parents towards accessing PT services for their child with ALL. Timing for initiation of PT services was one of the most variable areas identified in the survey. While some parents identified the maintenance phase as an appropriate time to initiate PT, others supported earlier introduction, reflecting how PT is commonly seen as a reactive service rather than as a standard component of care, despite evidence supporting early integration during active treatment phases [9]. Interviews helped us understand that the preferred timing to initiate the child’s PT intervention depended on each family’s situation and the child’s condition. This finding aligns with recent evidence in which some parents recommended introducing the service soon after diagnosis, while others preferred services when a specific need arose, and some reported no preference given the nature of the diagnosis [40]. Therefore, a tailored approach to initiating PT services (i.e., providing treatment) in children with ALL is recommended, taking into consideration the presence of physical deficits amenable to PT and families’ capacity to engage in rehabilitation services.

While survey results revealed that most parents were familiar with the benefits of PT services for their child, interview findings indicate that parents appreciate receiving education and knowledge upfront about possible changes in their child’s physical function. Educating families on the benefits of PT services and the signs and symptoms to look out for in their child, as well as building a relationship with the family prior to the initiation of a PT program, are essential to support early identification of deficits and a timely PT intervention [40,41]. Previous research identified that receiving information about their child’s care was one of the main needs of parents of children with cancer [40,42]. Access to information on the child’s condition as well as the involvement of social workers not only allows families to participate in healthcare activities but also to be aware of the options available and make informed decisions about their child’s care [42,43]. However, despite recent evidence highlighting the benefits of early access to rehabilitation services in childhood cancer survivors [44,45], families experience unique challenges and complexities resulting from the child’s medical treatment that may impact their participation in PT services. Similarly to results from our interviews, previous evidence has shown that uncertainty regarding the child’s cancer diagnosis, treatment, symptom management, and future is among the primary emotional needs parents face [42,46,47], which may impact parents’ decision to access PT services. Therefore, physical therapists must assess families’ capacity to start a PT program while at the same time respecting the families’ priorities regarding their child’s cancer care [48].

Interview findings show the crucial role that PT plays in the child’s recovery and in helping families navigate the child’s return to participation in physical activity. Therefore, improved access to PT services is crucial to optimize long-term outcomes in children with cancer [48]. Research has shown that parents of children with cancer face unmet needs, including but not limited to psychosocial, emotional, physical, informational, financial, educational, and spiritual [42,49]. Distance to PT services, time constraints, high number of hospital appointments, financial burden, negative impact on mental health, and limited accessibility were some of the common barriers parents experienced when accessing PT services, which are consistent with previous research studies exploring the barriers that families of children with cancer face when accessing healthcare services [29,42,43,50,51,52]. Access to pediatric oncology PT services can be improved when parents are provided with resources to minimize the unique barriers they face during their child’s cancer journey. As a multidisciplinary team, it is crucial to identify families’ needs from diagnosis, as this allows healthcare providers to timely respond to the needs [42], offering tailored resources, which can help minimize the cancer burden in families. Advancements in technology have created opportunities to explore innovative alternatives for the delivery of PT services, such as telehealth, which has been shown to be feasible in the pediatric oncology population [53,54]. Survey results revealed that factors such as a convenient location and the availability of virtual delivery would impact parents’ decision to access PT services. After further exploration of this topic through the interviews, findings are consistent with research evidence suggesting that offering alternatives to families, such as virtual PT services or connecting with PT locations in their communities [48,55], can help minimize some of the barriers families experience and may positively influence their decision to participate in PT services for their child.

Participants’ perspectives provided in the study are unique to the publicly funded healthcare system in Canada. Healthcare inequities related to socioeconomic status, insurance coverage, and health system structure can directly impact access to PT services and care [56,57,58,59,60,61]. Additionally, responses were likely influenced by the demographic characteristics of participants. For instance, most of the participants were parents of children under the age of 12, limiting our ability to gather more perspectives on the unique needs of families of adolescents with ALL. Likewise, a large proportion of participants were of European descent/White, with limited representation from other ethnic groups, which limits culturally diverse perspectives towards PT services for childhood cancer survivors. Therefore, findings should be interpreted within the context of the healthcare environment in which the study was conducted.

### 4.1. Actionable Strategies to Implement in Clinical Practice

Below, we outline five actionable strategies that align with the existing barriers and facilitators in our hospital setting. As an exploratory, single-center pilot study, these findings are intended to inform hypothesis generation and guide future research and implementation efforts rather than to provide generalizable or prescriptive recommendations for clinical practice.

*Guided by the Oncologist—Mapping each child and family’s path:* Children with cancer are more likely to engage in physical activity if it is recommended by their oncologist [50]. It is therefore recommended that oncologists introduce PT services at the beginning of cancer treatment (e.g., providing information about the service and the evidence base supporting its value) as a necessary and expected component of oncology care alongside chemotherapy and other interventions such as nutrition. While PT should be introduced early for all children with ALL, the timing of treatment may need to be flexible and tailored to the needs of the child and family—recognizing that each child’s “map” may look different. Evidence from early intervention models, including the Stoplight program [9], demonstrates that PT can be safely integrated during active treatment phases such as consolidation and supports benefit from mitigating functional decline [9,44]. Early education can include (1) how PT can support the child throughout the cancer journey, (2) potential changes in the child’s physical function that families should monitor and report, and (3) advice as to what activities could be beneficial for the child.

*Family education:* Families appreciate having access to educational materials that reinforce key information pertinent to their child’s care. Because not all caregivers are able to attend appointments, parents value being able to share and access information at their convenience. Providing educational materials through online platforms, using an engaging format and family-friendly language, is seen as helpful in improving understanding and in bridging the connection between the healthcare provider and family. In healthcare settings with limited resources, printed materials such as brochures, handouts, and posters are useful alternatives to help families access educational information.

*Tailored interventions:* PT should be tailored to the child’s interests, age, abilities, and physical condition to increase engagement and adherence [43,50,62]. Incorporating play, adapting exercise to focus on the child’s specific impairment, treatment phase, or energy level, and involving family participation can help maintain participation. Positive feedback and encouragement from the PT can further support motivation and adherence [50].

*Home-based activities:* Providing therapeutic interventions that can be incorporated into daily life activities can minimize parent burden as it optimizes their time, removes the stress from creating ideas [50], and allows the child to adopt new practices in a familiar environment. Involving family members and/or peers in therapeutic activities has a strong influence on the child’s motivation to engage in physical activities [43,50,51] and allows for social connection.

*Delivery modalities and locations:* Offering families options for how and where PT is delivered will allow parents to choose the delivery type that best accommodates their needs. Hybrid delivery (in-person and virtual) of PT services can help minimize common barriers such as distance to PT location, transportation and parking costs, commute time, and missing school and work. Virtual delivery allows for flexibility in scheduling given common fluctuations in the child’s health condition [50], and provides comfort to the child to exercise in a familiar space, where siblings can join. Facilitating referrals to community-based [48] or school PT services can support continuity of care without the need to travel long distances for specialized services. When a hospital-based PT service is preferred, coordinating appointments with the child’s hospital visits can reduce burden on families [29,48]. In cases where financial hardships are present and limit access to PT services (e.g., inappropriate health insurance coverage, travel expenses, lost family income), providing families with information about different sources of financial assistance from the government and charitable organizations may help alleviate and address some of these challenges [63].

### 4.2. Limitations

This study had several limitations. First, many of the study participants were parents of children undergoing the maintenance phase of ALL treatment, which can limit our ability to transfer the findings to families of children undergoing the first and middle phases of ALL treatment. Moreover, we did not collect data related to the timing of the maintenance treatment to examine if there were differences between those in the early versus later stages of maintenance treatment. However, this is consistent with the current clinic demographics given the relatively short duration of the initial ALL treatment phases (as compared to the maintenance phase) and a relatively limited number of new ALL diagnoses at the hospital in contrast to the number of children undergoing treatment during the later treatment phases. To try to address this limitation, we (1) ensured the study was offered to all eligible families whose children were undergoing or had completed ALL treatment, and (2) interviewed at least one parent of a child representing each of the main ALL treatment phases, including those off therapy. Second, although a convenience sample is appropriate in qualitative studies and may provide excellent insight about a phenomenon, it is possible that commonalities in this group of participants might have skewed the researcher’s perceptions about the phenomenon being studied. Consequently, the researcher’s credibility to the interpretation of findings may be limited to a specific study context [34]. Third, given the relatively small sample size and the unique nature of the Canadian healthcare system, generalization of results will be limited, and translation of findings should be exerted with caution. Fourth, although all eligible parents of children with ALL were approached with information about the study, selection bias may have occurred, as families with higher motivation may have been more likely to consent to participate.

Results from this study served to inform the design of a 12-week hybrid, in-person, and online proof-of-concept PT program for children with ALL [64].

## 5. Conclusions

Understanding parents’ perspectives towards PT for children with ALL is essential to help improve the capacity to access services. Parents of children with ALL value PT and are willing to engage in PT if the needed resources are in place. Flexibility in service delivery, availability of virtual PT options, incorporation of therapeutic interventions into daily activities, and coordination of community and school-based PT services can enhance access and sustain family participation in PT programs.

## Figures and Tables

**Table 1:** Survey questions.

COM-B Model	TDF Domains	Questions
Capability	Knowledge	How familiar are you with the benefits of physiotherapy for children with cancer?
	Memory, attention and decision processes	Would you consider accessing physiotherapy services for your child if they were having difficulty with their physical ability (e.g., balance issues, difficulty walking or climbing stairs)?
	Nature of Behaviors	Is physiotherapy something you would consider for your child while undergoing cancer treatment? If your child were to take part in a physiotherapy program that aimed to help reduce side effects and maintain their physical ability, when would you prefer this type of program to start?
	Behavioral Regulation	What are some of the specific physical challenges that may prompt you to seek physiotherapy for your child?
	Skills	How easy or hard do you think it would be for you to access physiotherapy services for your child?
Motivation	Beliefs about capabilities (self-efficacy)	How confident are you in deciding if your child needs physiotherapy treatment?
	Emotion	If your child was having issues with activities such as walking or getting dressed and needed to be referred to physiotherapy services, how would you feel about it?
	Social/professional role and identity (self-standards)	How do you feel you can best support your child’s physiotherapy (if needed)? Who would you consider responsible for referring your child to physiotherapy services?
	Beliefs about Consequences (anticipated outcomes/attitudes)	If your child was having an issue such as pain, difficulty walking or muscle weakness, how do you think physiotherapy could help? In your opinion, would the benefits of physiotherapy for your child justify the costs for you (including time)?
	Motivation/Goals (intentions)	How easy or difficult would it be for your child to do exercises at home (e.g., a home exercise program assisted by a parent/caregiver)? How easy or difficult would it be for you to support your child in doing exercises at home (e.g., a home exercise program assisted by you)?
Opportunity	Environmental context and resources (environmental constraints)	What resources would need to be in place for you to access physiotherapy services for your child? If your child needs physiotherapy, what factors would influence your decision to access these services?
	Social Influences (norms)	Would others (e.g., family or other support persons) be supportive of your child accessing physiotherapy?

Survey questions mapped into the Capability, Opportunity, Motivation—Behavior Change (COM-B) Model and the Theoretical Domains Framework (TDF) domains.

**Table 2:** Summary of participant characteristics.

Demographics	Overall N *=* 20, 100%
**Biological Sex/All cisgender (n, %)**	
Female	15 (75%)
Male	5 (25%)
**Marital Status (n, %)**	
Never Married	1 (5%)
Married	17 (85%)
Common Law	1 (5%)
Separated	0 (0%)
Widowed	0 (0%)
Divorced	1 (5%)
**Education (n, %)**	
Some High School	2 (10%)
Completed High School	3 (15%)
Some University/College	3 (15%)
Completed University/College	11 (55%)
Completed Graduate School	1 (5%)
**Current Employment Status (n, %)**	
Retired	1 (5%)
Disability	1 (5%)
Part Time	4 (20%)
Homemaker	5 (25%)
Full Time	8 (40%)
Temporarily Unemployed	1 (5%)
**Annual Family Income (n, %)**	
<20,000	1 (5%)
20–39,999	1 (5%)
40–59,999	2 (10%)
60–79,999	4 (20%)
80–99,999	2 (10%)
>100,000	8 (40%)
Prefer not to answer	2 (10%)
**Type of Residential Area (n, %)**	
Edmonton Metropolitan Area	11 (55%)
Alberta/NWT rural residence	6 (30%)
Alberta/NWT remote residence	3 (15%)
**Living in the Same Residence as the Child (n, %)**	
Yes	19 (95%)
No	1 (5%)
**Ethnic Origin or Ancestry (n, %)**	
European Descent/White	14 (70%)
Aboriginal	1 (5%)
East/Southeast Asian	3 (15%)
Latin/Central and South American	1 (5%)
Not reported	1 (5%)
**Child’s Age at the Time of Survey (Mean, SD, range)**	
Age, years	7.3 ± 2.76 (4–14 years)
**Child’s Chemotherapy Treatment Phase (n, %)**	
Consolidation	2 (10%)
Maintenance	16 (80%)
Off-therapy	2 (10%)

Demographic characteristics of parents and their child diagnosed with acute lymphoblastic leukemia.

## Data Availability

The data presented in this study are available on the tables, figures, and Supplementary Materials.

## Supplementary Materials

The following supporting information can be downloaded at https://www.mdpi.com/article/10.3390/curroncol33010060/s1: Document S1: Survey. Document S2: Semi-structured interview questions.

## Abbreviations

The following abbreviations are used in this manuscript:

ALL	Acute lymphoblastic leukemia
COM-B	Capability, Opportunity, Motivation -Behavior Change Model
PT	Physical therapy
TDF	Theoretical Domains Framework
