# Supplementary material for: Parent Perspectives on Physical Therapy for Their Child with Acute Lymphoblastic Leukemia: The Light at the End of the Tunnel
Source: Curr Oncol. 2026 Jan 20;33(1):60. doi: 10.3390/curroncol33010060 (PMC12839667; doi:10.3390/curroncol33010060)
Supplement: Supplementary file 1 [file curroncol-33-00060-s001.zip › S2 - Semi-structured interview questions.pdf]

Semi-structured interviews with parents of children with acute lymphoblastic leukemia

Format: ☐ Zoom ☐ In-person

Welcome statement:

Hi __(name of parent)__! Thank you very much for agreeing to take part in this interview. The purpose of this session is to understand better your thoughts on physiotherapy services for your child based on the answers provided on the survey that you kindly completed a couple of months ago.

I would like to start by sharing some considerations of this interview, please feel free to interrupt me at any time if you have any questions…

1. Your participation in this interview is entirely voluntary and you can choose to end your participation at any time for any reason.
2. This interview will take between 40 to 60 minutes; however, if you wish you expand on your answers or provide added information, we can extend the session.
3. You can stop this interview at any time and resume when you feel ready.
4. We can take as many breaks as you need.
5. This interview will be audio recorded and transcribed.
6. To protect your privacy and confidentiality, your name will not be included. For today's interview, you can choose a fake or alternate name. We will only include a study ID on the transcriptions and no identifying information will be used when summarizing the research findings.
7. Please speak as openly as you feel comfortable. If there are sensitive topics you do not wish to answer, you are welcome to pass on these questions.
8. You can skip questions and we can come back to a topic if you wish to expand or change anything you said.
9. Please remember that the information shared in this interview is confidential and will not in any way impact you or your child's health care nor will it be shared with the healthcare team.

Do you have any questions or comments before we start?

Interviewer: Let the participant know that the recording will start.

After pressing "record"…

Before we start the interview, could you please confirm again that you agree to take part in this interview and be recorded?

Thank you!

QUESTIONS

BENEFITS OF PT AND PERSPECTIVES OF THE SERVICE

1). I would like to start by hearing more about your thoughts on the benefits of physiotherapy for your child.

> **1.1** Based on what I hear, you have pointed out important benefits such as __(provide examples of benefits discussed)__. Can you provide me with some examples of how physiotherapy has helped your child during and after* (*if applicable) treatment?
>
> **1.2.** From our survey results we have found that many parents believe that the benefits of physiotherapy for their child justifies the time and costs of therapy. Does this finding resonate with you? Why?

2). Our survey results revealed that all parents would consider accessing physiotherapy if their child presents with any problems that require therapy; however, over 50% of parents said they would prefer to start physiotherapy for their child during the maintenance phase of chemotherapy. Can you tell me your thoughts on when it may be best to start physiotherapy and why?

> **2.1.** On that note, when during your child's treatment would you have preferred a discussion on physiotherapy services, that may include providing some initial education on the value/ and how to access our services?

3). Now I would like to know your thoughts on the ideal physiotherapy treatment location and environment for your child. You can talk about location (*e.g.,* hospital, community, home), space (*e.g.,* equipment), staff (*e.g.,* disciplines available), costs (*e.g.,* parking), etc.

> **3.1** From what I hear, you envision a __(recall details)__ as an ideal physiotherapy space for your child!
>
> > **3.1.1** *(If not mentioned)* How do you feel about your child exercising with other children at a kids' gym space or indoor playground?
> >
> > **3.1.2** *(If not mentioned)* What are your thoughts on a program offered at the hospital?
> >
> > **3.1.3** What are your thoughts on participating in a physiotherapy program for your child following the oncology appointment at the hospital?

BARRIERS AND FACILITATORS

4. Now I would like to talk about the support and resources that would need to be in place for you, as a parent, to access physiotherapy services for your child. Our survey results revealed that parents identified 'a convenient/ accessible location' as the most common factor when considering accessing the physiotherapy for their child…

4.1. How do you think we can best accommodate this need/ preference?

4.2 Almost 50% of parents indicated they would consider accessing the service if there were more options for physiotherapy delivery such as virtual programs. What are your thoughts on participating in, or accessing virtual (online) physiotherapy for your child?

4.2.1 From what I hear, you think virtual physiotherapy is __(describe main points mentioned)__.

If barriers were mentioned… (if applicable)

- How do you think we can help address __(describe barrier(s))__ for you and your child to participate in a virtual physiotherapy program?

*If barriers were **not** mentioned… (if applicable)*

- (*if not mentioned*) What are the concerns that come to your mind when you think about your child taking part in an online physiotherapy program?

- Our survey results showed that opinions were divided, and while many parents indicated that it would be easy to support their child's participation in a home program given the availability of time, space, and equipment; others shared some issues such as limited child's motivation without the support of a therapist, limited time, and possible distractions at home. How do you think we can best support parents to accommodate for these barriers?

VIRTUAL PLATFORM

5). Now I am going to share with you a preliminary version of the virtual platform we are creating to offer online physiotherapy services for children.

5.1 I would like to hear your thoughts on the services available on this platform:

- *General:* What are your thoughts on this approach/ direction for services? What did you like, dislike or would like to improve?

- *Educational materials:* Do you think the amount of written content vs graphics is appropriate? Are there any topics you would like to have included on this platform?

- *Physical activity resources:* Do you prefer a video demonstrating the exercise or would a photo with text and ideas on how to incorporate exercises into day-to-day activities be enough to support your child's care?

OPTIONAL
Questions to former participants of the other programs/ studies:

6). Have you participated in any other physical activity or exercise programs for your child?

> **6.1.** IF yes, can you tell me about the program, what you liked, what worked or did not work for you/ your child?
