# Supplementary material for: Parent Perspectives on Physical Therapy for Their Child with Acute Lymphoblastic Leukemia: The Light at the End of the Tunnel
Source: Curr Oncol. 2026 Jan 20;33(1):60. doi: 10.3390/curroncol33010060 (PMC12839667; doi:10.3390/curroncol33010060)
Supplement: Supplementary file 1 [file curroncol-33-00060-s001.zip › S1 - Survey.pdf]

Physiotherapy Needs of Children - Survey

Dear participant,

Thank you for taking the time to provide feedback!

This survey will take approximately 15-20 minutes to complete and comprises questions about your views towards physiotherapy services for children undergoing treatment for acute lymphoblastic leukemia.

You are allowed to stop the survey at any time and return to it later. In order to save your answers and continue where you left off, you must scroll down to the end of the page and click the "Save and Return Later" button.

For any questions or concerns, please contact us:

- [redacted]

1. How familiar are you with the benefits of physiotherapy for children with cancer?

Not at all — Some — Very much

(Place a mark on the scale above)

2. Would you consider accessing physiotherapy services for your child if they were having difficulty with their physical ability (e.g., balance issues, difficulty walking or climbing stairs)?

- ◯ Yes
- ◯ No
- ◯ Don't know

3. How confident are you in deciding if your child needs physiotherapy treatment?

- ◯ Very much
- ◯ Somewhat
- ◯ Undecided
- ◯ Not really
- ◯ Not at all

4. Is physiotherapy something you would consider for your child while undergoing cancer treatment?

- ◯ Yes
- ◯ No
- ◯ Don't know

5. If your child was having issues with activities such as walking or getting dressed and needed to be referred to physiotherapy services, how would you feel about it?

Upset/ concerned — Neutral — Calm/confident

(Place a mark on the scale above)

6. If your child were to take part in a physiotherapy program that aimed to help reduce side effects and maintain their physical ability, when would you prefer this type of program to start?

(Please select the answer that best describes your preference, or select OTHER and provide further details)

- ◯ During the Induction phase of chemotherapy
- ◯ During active treatment (consolidation/intensification phases)
- ◯ During the maintenance phase of chemotherapy
- ◯ After completion of chemotherapy
- ◯ Other

Other (please provide details)

____

7. What are some of the specific physical challenges that may prompt you to seek physiotherapy for your child?

(Please select all that apply. You may click on "Other" to include additional responses not listed).

- ☐ Pain
- ☐ Muscle weakness
- ☐ Trouble going up/down the stairs
- ☐ Challenges with balance (e.g., falling)
- ☐ Numbness, tingling, or other sensory problems
- ☐ Changes to how they walk (e.g., limping or heavy feet)
- ☐ Not able to keep up with friends/siblings (e.g., slower runner)
- ☐ Limitations in daily activities (e.g., buttoning clothes, holding a pencil, playing)
- ☐ Other

Other (please provide details) ____

8. How easy or hard do you think it would be for you to access physiotherapy services for your child?

- ◯ Very hard
- ◯ Neutral
- ◯ Very easy
- ◯ Don't know

9. What resources would need to be in place for you to access physiotherapy services for your child?

(Please select all that apply. You may select "Other" to include additional responses not listed).

- ☐ Public or private healthcare coverage for costs of physiotherapy services
- ☐ Coverage to pay for indirect/other costs (e.g., parking, childcare, time off work)
- ☐ Convenient/accessible location
- ☐ Time
- ☐ Other (please describe)

Other (please provide details) ____

10. How do you feel you can best support your child's physiotherapy (if needed)?

(Please select all that apply. You may click on "Other" to include additional responses not listed).

- ☐ Helping them with exercises at home
- ☐ Arranging physiotherapy appointments
- ☐ Providing encouragement
- ☐ Don't know
- ☐ Other

Other (Please describe) ____

11. Who would you consider responsible for referring your child to physiotherapy services?

(Please select all that apply. You may click on "Other" to include additional responses not listed).

- ☐ Oncologist
- ☐ Nurse
- ☐ Family Doctor
- ☐ Teacher at school
- ☐ Don't know
- ☐ Other

Other (Please describe) __________________________________

12. If your child was having an issue such as pain, difficulty walking or muscle weakness, how do you think physiotherapy could help? ____

13. In your opinion, would the benefits of physiotherapy for your child justify the costs for you (including time)?	◯ Yes<br>◯ No<br>◯ Don't know
14. If your child needs physiotherapy, what factors would influence your decision to access these services?<br><br>(Please select all that apply. You may click on "Other" to include additional responses not listed).	☐ Personal income<br>☐ Distance to physiotherapy service<br>☐ Expertise of physiotherapist<br>☐ Research evidence supporting physiotherapy interventions<br>☐ Availability of options for physiotherapy delivery: home-based, virtual, in-person<br>☐ Other
Other (Please describe)	__________________________________
15. How easy or difficult would it be for your child to do exercises at home (e.g., a home exercise program assisted by a parent/caregiver)?	Very easy Neutral Very difficult<br>*(Place a mark on the scale above)*
Please explain why:	____
16. How easy or difficult would it be for you to support your child in doing exercises at home (e.g., a home exercise program assisted by you)?	Very easy Neutral Very difficult<br>*(Place a mark on the scale above)*
Please explain why:	____
17. Would others (e.g. family or other support persons) be supportive of your child accessing physiotherapy?	◯ Yes<br>◯ No<br>◯ Don't know
